# Increased expression of ubiquitin C-terminal hydrolase L1 in astrocytomas of ascending grades

**DOI:** 10.1097/MD.0000000000034132

**Published:** 2023-06-30

**Authors:** Emir Kaan İzci, Fatih Keskin, Fatih Erdi, Bulent Kaya, Yasar Karatas, Bahadir Feyzioglu, Siddika Findik, Erdal Kalkan, Hasan Esen, Önder Guney

**Affiliations:** a Department of Neurosurgery, Baskent University Konya Application and Research Hospital, Konya, Turkey; b Department of Neurosurgery, Meram Faculty of Medicine, Necmettin Erbakan University, Konya, Turkey; c Department of Neurosurgery, Medova Hospital, Konya, Turkey; d Department of Neurosurgery, Afyon Park Hospital, Afyon, Turkey; e Department of Microbiology, Meram Faculty of Medicine, Necmettin Erbakan University, Konya, Turkey; f Department of Pathology, Meram Faculty of Medicine, Necmettin Erbakan University, Konya, Turkey.

**Keywords:** astrocytoma, expression, glioblastoma, ubiquitin carboxy-terminal hydrolase-L1

## Abstract

The ubiquitin-proteasome pathway controls the monitoring and degradation of important proteins and is involved in several cellular processes, such as development, differentiation, and transcriptional regulation. Recent evidence has shown that ubiquitin carboxy-terminal hydrolase-L1 (UCH-L1), a member of the deubiquitinating enzyme family that removes ubiquitin from protein substrates, is overexpressed in many types of cancer.

**Aim:**

This study thus examined the expression of UCH-L1 in human astrocytoma tissues.

**Material and methods:**

Formalin-fixed, paraffin-embedded astrocytoma samples were obtained from 40 patients, after which histopathological examination, typing, and grading were performed. The study group included 10 histologically normal brain tissues, which served as the control group, and 10 WHO grade II, 10 WHO grade III, and 10 WHO grade IV (glioblastoma) samples. Normal brain tissue samples were obtained from the histologically normal, non-tumoral portion of the pathology specimens. UCH-L1 expression was evaluated using quantitative reverse transcription-polymerase chain reaction and immunohistochemistry.

**Results:**

Astrocytoma tissues exhibited higher UCH-L1 expression compared to the control group. UCH-L1 overexpression increased significantly together with the increase in astrocytoma grades (from II to IV).

**Conclusion:**

UCH-L1 could be a good diagnostic and therapeutic marker for determining astrocytoma development and progression.

## 1. Introduction

Astrocytomas are the most common intrinsic central nervous system neoplasms, particularly in adults. They are the most aggressive primary brain tumors and those with the poorest vital prognosis.^[1]^ The knowledge about the molecular basis of astrocytoma development and progression has increased significantly, especially in the last decade. They are graded in the sixth version of World Health Organization (WHO) Classification of Tumors of the Central Nervous System (CNS) as the international standard for the classification of brain and spinal cord tumors.^[2]^

Ubiquitin carboxy-terminal hydrolase-L1 (UCH-L1) belongs to the deubiquitinating proteases family that removes ubiquitin from protein substrates and precursor proteins. Deubiquitinating proteases are involved in abundant biological processes in cells like development, differentiation, and transcriptional regulation.^[3]^

Besides its biological roles, UCH-L1 was suggested to be a crucial regulator of invasion and metastasis in various types of cancer including neuroblastoma,^[4]^ pancreatic endocrine tumors,^[5]^ and non-small cell lung cancer.^[6]^ Previous studies showed the constitutive expression of UCH-L1 in human neural cell lines.^[7]^ Studies of traumatic brain injury also showed that UCH-L1 expression was increased according to the severity of trauma and that it indicates a poor outcome.^[8]^

In this study, we examined the expression of UCH-L1 in human astrocytoma tissues with quantitative reverse transcription polymerase chain reaction (qRT-PCR) and immunohistochemistry for the first time in the literature.

## 2. Methods

### 2.1. Patient group

Surgically resected astrocytoma specimens from 40 patients who underwent surgery at our institute between 2005 and 2017 were obtained from the pathology archive. The specimens had been fixed in 4% phosphate-buffered formaldehyde, after which they had been processed into paraffin blocks. Histopathological examination, typing, and grading of the hematoxylin and eosin-stained slides were performed.^[5]^ The study group included 10 histologically normal brain tissue samples, which served as the control group, 10 WHO grade II, 10 WHO grade III, and 10 WHO grade IV (glioblastoma) samples. Normal brain tissue samples were obtained from the histologically normal non-tumoral portion of the pathology specimens. None of these patients were administered radiotherapy, chemotherapy, or immunotherapy prior to their surgery.^[1]^ Informed consent and ethical approval for the study were obtained. Ethical approval for this study was obtained from the relevant institutional review board with date 05/01/2018 and number 2018/1148 (Suleyman Demirel University, Meram Medical School, Ethics Committee of Searchs without Medical Equipments and Medicine), ensuring that the research protocol adheres to established ethical guidelines and safeguards the rights and welfare of the participants.

### 2.2. RNA isolation and qRT-PCR

Ribonucleic acid (RNA) isolation and qRT-PRC evaluation were completed in a standard fashion. Total RNA was isolated from formalin-fixed, paraffin-embedded (FFPE) tissues using the High Pure FFPET RNA Isolation Kit (Roche Applied Science, cat. 6868517, Basel, Sweeden) according to the manufacturer’s instructions. The Transcriptor First Strand c-DNA Synthesis Kit (Roche Applied Science) was used to generate c-DNA from RNA, according to the manufacturer’s instructions; 10 μL total RNA was used for amplification.^[9]^ Amplification reactions were set up in a reaction volume of 20 μL using the LightCycler 480 PCR Master Mix (Roche Applied Science, cat. 04707494001). PCR primaries and TaqMan probes (Roche Applied Science, cat. 05532957001) were synthesized and preoptimized. qRT-PCR was performed using PCR primaries (RT-PCR TaqMan® assays Catalog No; [ID: 108862 UCHL1 H.sapiens PARK5, PGP 9.5, PGP9, PGP95, Uch-L 1100121980] and [ID: 104092, RN18S1 H.sapiens 100121999]) and TaqMan probes in the LightCycler 480 II system (Roche Applied Science). The following primers were used: UCHL1-F 50-CCTGAAGACAGAGCAAAATGC-30; UCHL1-R 50-AATGGAAATTCACCTTGTCATCT-3. Amplification was performed for 40 cycles at 95°C for 10 seconds, followed by 60°C for 30 seconds, and 72°C for 1 second.^[9]^ Quantification was achieved using the comparative CT method, normalizing against the 18S ribosomal RNA (18S) gene. The expression levels in tumor tissues relative to the non-tumor controls were assessed using the 2 −ΔΔCt method.^[9]^ Briefly, the threshold cycle (Ct) of fluorescence for each sample was determined. ΔCt indicates the difference in expression levels between UCH-L1 and 18S(ΔCt = Ct UCHL1 − Ct18S), and ΔΔCt indicates the difference in the ΔCt value between the tumor tissue and the control (ΔΔCt = ΔCttumor − ΔCtcontrol). The 2^−ΔΔCt^ value (fold-value) was calculated.

### 2.3. Immunohistochemistry

Paraffin-embedded tissue samples were extracted and sectioned (4 µm thickness), and then mounted onto positively charged slides (Objektträger, Rhede, Germany).

The procedure was performed using an automated avidin-biotin system (Ventana Benchmark XT, Ventana Medical Systems, Tucson, AZ) employing the PGP 9,5 antibody (1:100, Abcam, Anti-PGP 9.5 antibody ab53057, rabbit polyclonal antibody, Waltham, MA).

Cytoplasmic immunoreactivity patterns were evaluated and categorized as negative 0, weak 1, moderate 2, or intense 3 positives.^[10]^

### 2.4. Statistical analysis

Statistical analyses were performed using SPSS for Windows 18.0 (SPSS Inc., Chicago, IL). The data were expressed as median or mean ± standard deviation (SD). The statistical comparisons of the groups were performed by the Kruskal–Wallis test. The significant results for pairwise comparisons were analyzed by the Mann–Whitney *U* test with Bonferroni correction. A *P* value of less than .05 was considered statistically significant.

## 3. Results

### 3.1. qRT-PCR results

The mean changes in the mRNA level of UCH-L1 in astrocytoma, relative to control tissue, were 52.06 ± 17.14 for grade II, 233.26 ± 18.37 for grade III, and 658.50 ± 84.44 for grade IV. The difference in the groups was found to be statistically significant (*P* < .001). All results of the groups were significantly different from each other (*P* < .05).

**Figure 1.**
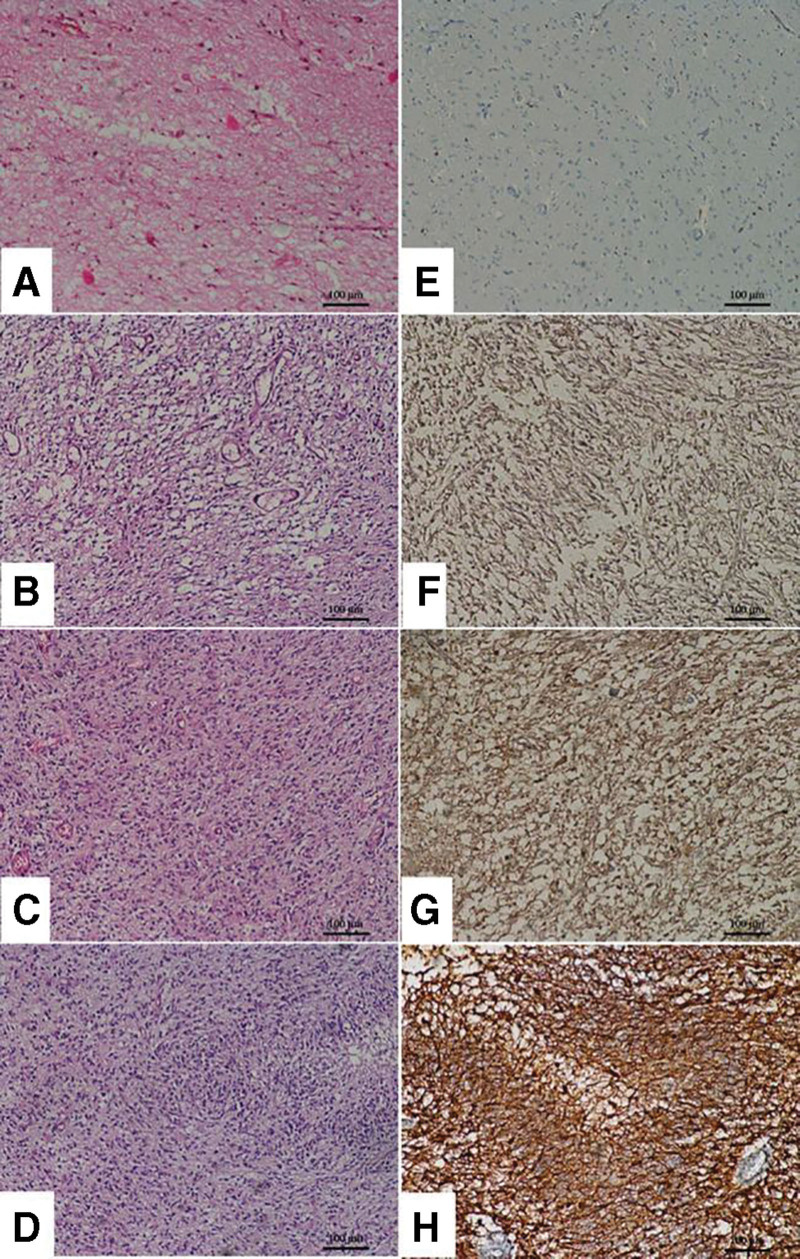
qRT-PCR results of UCH-L1 expression of astrocytoma tissues. A. 18S CT values, B. UCH-L1 CT values, C. Normalized UCH-L1 mRNA expression relative to control values. .

The minimum and maximum n-fold changes in UCH-L1 expression of astrocytoma were 2.77–235.57 for grade II, 45.89–436.55 for grade III, and 40.50–1398.83 for grade IV. qRT-PCR results were summarized in Table 1. Moreover, the qRT-PCR results were visualized in Figure 1.

**Table 1:** Fold change in expression of UCHL1 gene relative to the reference gene 18S in the control group and different grade astrocytomas.

Sample groups	18S Ct	UCHL1 Ct	ΔCt (Avg. UCHL1 − Avg. 18S)	ΔCt (Avg. ΔCt − Avg. ΔCt control ± SD)	Normalized UCHL1 mRNA expression relative to control 2^ΔΔCt^ ± SD (min–max)	*P*
Control	18.5	30.02	11.52	0	1.0 ± 0.0^a,b,c^ (0.90–1.13)	
Grade II	22.16	29.64	7.48	−4.04 ± 4.1	52.06 ± 17.14^a,d,e^ (2.77–235.57)	<.001*
Grade III	24.07	28.11	4.05	−7.47 ± 4.2	233.26 ± 18.37^b,d,f^ (45.89–436.55)	
Grade IV	23.88	26.55	2.67	−8.85 ± 6.4	658.50 ± 84.44^c,e,f^ (40.50–1398.83)	

Superscript letters denote the significant pairwise comparisons at 0.008 due to Bonferroni correction.

UCH-L1 = ubiquitin carboxy-terminal hydrolase-L1, SD = standard deviation.

* Significant at .05 level according to Kruskal–Wallis test.

### 3.2. Immunohistochemistry results

**Figure 2.**
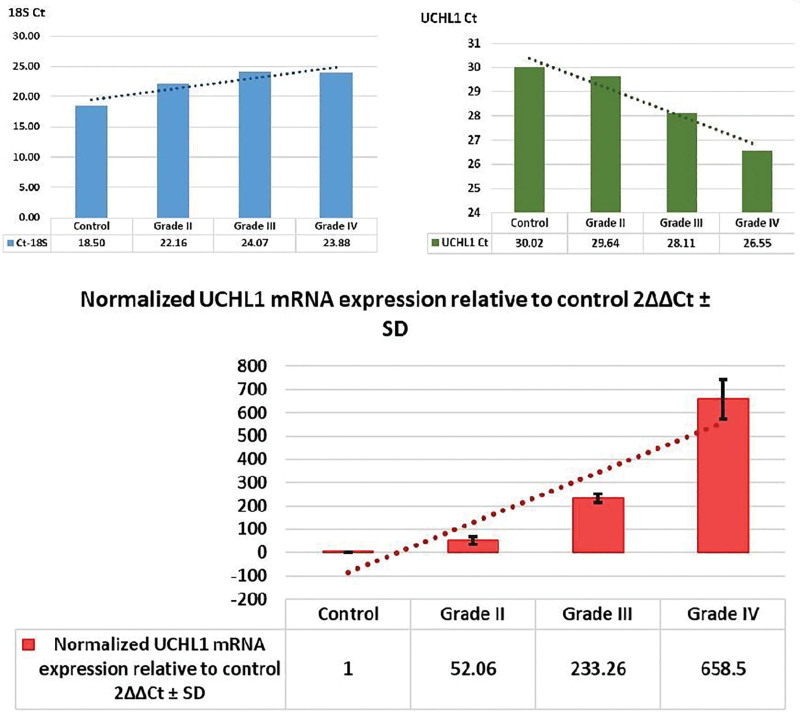
H&E and UCH-L1 immunostaining images. Representative images of control and grade II-IV astrocytoma. Hematoxylin and eosin (H&E) staining and immunohistochemistry images for UCH-L1. Increased staining intensity for UCH-L1 can be seen according to ascending grades of astrocytoma tissues. A. Histologically normal cerebral tissue (H&E, × 100), B. Grade II astrocytoma tissue (H&E, × 100), C. Grade III astrocytoma tissue (H&E, × 100), D. Grade IV astrocytoma tissue (H&E, × 100), E. Negative expression of UCH-L1 in histologically normal brain tissue, F. Weak UCH-L1 expression in grade II astrocytoma, G. Moderate UCH-L1 expression in grade III astrocytoma h. Intense UCH-L1 expression in a glioblastoma sample. UCH-L1 = ubiquitin carboxy-terminal hydrolase-L1.

All astrocytoma tissues showed statistically significant high cytoplasmic immunoreactivity compared to the control group (*P* = .006). Immunostaining positivity increased statistically significantly (*P* < .008) according to ascending grades from grade II to IV. Representative hematoxylin and eosin and UCH-L1 immunostaining images are presented in Figure 2. The detailed descriptive data obtained from immunohistochemistry was presented in Table 2.

**Table 2:** Results of immunohistochemistry.

Group	Mean ± SD	Median	Min–Max	*P*
Control	0.22 ± 0.45^a^	0	0–1	
Grade II	1.23 ± 1.02^a,d^	1	0–3	.006*
Grade III	1.67 ± 1.04^b^	2	0–3	
Grade IV	2.11 ± 0.73^c,d^	2	1–3	

Superscript letters denote the significant pairwise comparisons at 0.008 due to Bonferroni correction.

SD = standard deviation.

* Significant at .05 level according to Kruskal–Wallis test.

## 4. Discussion

The autophagy-lysosome pathway and the ubiquitin-proteasome (UPS) pathway are 2 important pathways that control protein monitoring and degradation.^[11]^ Deubiquitinating enzymes of the UPS are a novel concern for the development of new cancer therapeutics due to their frequent dysregulation in multiple cancer types.^[11]^ The main subject of this study, UCH-L1, belongs to the family of deubiquitinating enzymes which remove ubiquitin from protein substrates.^[11]^

Recent evidence has verified the overexpression of UCH-L1 in many types of cancer including but not limited to acute lymphoblastic leukemia, non-small cell lung cancer, neuroblastoma, pancreatic, prostate, and medullary thyroid cancers.^[11]^ It was also shown that UCH-L1 is a key regulator of tumor invasion and metastasis.^[12]^

Studies concerning the molecular basis of astrocytoma development and progression increased significantly, especially in recent years. In the present study, we analyzed the expression of UCH-L1 in surgically resected astrocytoma tissues from several grades using qRT-PCR and immunohistochemistry methods for the first time in the literature. Our results showed that UCH-L1 was overexpressed in astrocytoma tissues when compared with a control group acquired from the non-tumoral portion of the pathology specimens. UCH-L1 overexpression was significantly increased according to ascending grades of astrocytoma samples from grade II to IV.

Selective degradation of proteins by the UPS pathway is a critical determinant for providing cellular homeostasis. The UPS degradation pathway plays an essential role in both the up-regulation of cell proliferation and the down-regulation of cell death in human cancer cells.^[13]^

Targeting the ubiquitin-proteasome system in hematological malignancies^[14]^ and solid tumors^[15]^ has attracted great attention with several rates of success reported. Previous studies showed the clear distribution of UCH-L1 in the brain which constitutes up to 5% of total neural proteins.^[15]^ It was shown that UCH-L1 is not essential for neuronal development but it is precisely necessary for the preservation of axonal integrity. Dysfunction of UCH-L1 was also implicated in the pathogenesis of various neurodegenerative diseases.^[15]^

The proteasome complex modulates the activity of important substrates which administrate cell proliferation, apoptosis, angiogenesis, and metastasis, such as tumor suppressor protein p53 and transcription factor nuclear factor kappa-B.^[3]^ The capacity of the proteasome for impairment of tumor-suppressing and proapoptotic protein targets is known to be dysregulated in many human malignancies, providing the reason for choosing the proteasome as a target for cancer treatment.^[15]^

Areeb et al^[16]^ investigated the inhibitory effects of proteasome antagonist carfilzomib on glioblastoma cell proliferation, migration, and invasion and reported that the second-generation proteasome inhibitor carfilzomib should be considered a potential therapeutic agent for the treatment of glioblastoma.

The role of UCH-L1 in oncogenesis and specific targets in the deubiquitinating process have yet to be determined.^[8]^ According to our literature search, we could not find any study which investigates the UCH-L1 expression profile in astrocytoma. UCH-L1 regulates many signaling pathways and could induce cancer cell invasion and metastasis due to its post-translational deubiquitinating activity.^[11]^

Astrocytoma constitutes a major part of the human intrinsic central nervous system neoplasms. The most common form of this neoplasm is glioblastoma which currently has no curative treatment. In this study, we found overexpression of UCH-L1 in astrocytoma tissues according to ascending grades. We suggest that UCH-L1 might be a good diagnostic and therapeutic target for checking astrocytoma development and progression.

## Author contributions

**Conceptualization:** Emir Kaan İzci, Fatih Keskin.

**Formal analysis:** Fatih Erdi, Bulent Kaya, Bahadir Feyzioglu.

**Investigation:** Yasar Karatas, Siddika Findik.

**Methodology:** Emir Kaan İzci, Erdal Kalkan, Önder Güney.

**Software:** Hasan Esen.

**Validation:** Fatih Erdi, Bulent Kaya.

**Visualization:** Fatih Keskin, Önder Güney.

**Writing – original draft:** Yasar Karatas, Bahadir Feyzioglu, Hasan Esen.

**Writing – review & editing:** Emir Kaan İzci.
